# NAUTICA: classifying transcription factor interactions by positional and protein-protein interaction information

**DOI:** 10.1186/s13062-020-00268-1

**Published:** 2020-09-16

**Authors:** Stefano Perna, Pietro Pinoli, Stefano Ceri, Limsoon Wong

**Affiliations:** 1 Dipartimento di Elettronica, Informazione e Bioingegneria (DEIB), Politecnico di Milano, Via Giuseppe Ponzio 34/5, 20133 Milan, Italy; 2 National University of Singapore, Singapore, Singapore

**Keywords:** Transcription factors, Interaction classification, Protein−protein interactions, TF-TF competition, Data-driven analysis

## Abstract

**Background:**

Inferring the mechanisms that drive transcriptional regulation is of great interest to biologists. Generally, methods that predict physical interactions between transcription factors (TFs) based on positional information of their binding sites (e.g. chromatin immunoprecipitation followed by sequencing (ChIP-Seq) experiments) cannot distinguish between different kinds of interaction at the same binding spots, such as co-operation and competition.

**Results:**

In this work, we present the Network-Augmented Transcriptional Interaction and Coregulation Analyser (NAUTICA), which employs information from protein-protein interaction (PPI) networks to assign TF-TF interaction candidates to one of three classes: competition, co-operation and non-interactions. NAUTICA filters available PPI network edges and fits a prediction model based on the number of shared partners in the PPI network between two candidate interactors.

**Conclusions:**

NAUTICA improves on existing positional information-based TF-TF interaction prediction results, demonstrating how PPI information can improve the quality of TF interaction prediction. NAUTICA predictions - both co-operations and competitions - are supported by literature investigation, providing evidence on its capability of providing novel interactions of both kinds.

**Reviewers:**

This article was reviewed by Zoltán Hegedüs and Endre Barta.

## Background

The classification of interactions between transcription factors (TFs) is foundational to the study of regulatory modules, i.e. groups of TFs implicated in the regulation of the same genes / transcriptional pathways. Classification based on localized binding-site information alone presents significant challenges, due to the confounding effect of intervening factors and the fact that some interactions happen only in the regulatory regions specific to certain genes or in noncoding area.

It is challenging to infer the precise nature of the interactions between two or more TFs, as they are dependent on their target, the cellular context in which the study is performed, and so on [1]. Transcription factors can compete to bind to a shared partner, compete for the same binding spots, or cooperate to coregulate some genes (and not others). Also, an investigation of all possible interactions between transcription factors (even for small genomes) is combinatorial in nature, and the cost of said wet lab experiments grows with the number of potential candidates.

In our previous work, we developed the Transcriptional Interaction and Coregulation Analyzer (TICA) [2], a tool for the discovery of new interaction candidates from human ChIP-Seq datasets using statistical inference, based on the relative positioning of the two potential interactors’ binding sites determined from ChIP-Seq experiments. One of the challenges of TICA is that it cannot discern the nature of the interaction itself. The positional nature of a TICA prediction only ensures the presence of physical interaction at a molecular level without inferring the functional nature of the interaction itself; other TF-TF interaction prediction tools (TACO [3], CENTDIST [4], etc.) based purely on binding site information derived from e.g. ChIP-Seq peaks and/or TF binding motifs share this same limitation.

Intuition suggests that a high number of shared protein-protein interactions is indicative of cooperative behaviour, while the reverse indicates competition for shared partners or no interaction at all. To quantify this, we could use the number of shared interactions in a reference protein-protein interaction (PPI) network, viz. BioGRID [5], as a measure of co-operation between transcription factors. However, it is not straightforward to classify interactions as cooperative or competitive solely by this measure. Consider for example the following cases: *HDAC1* and *E2F1* - evidence presented by Doetzlhofer et al. [6] indicates that *HDAC1* and *E2F1* compete for binding to the C terminus of transcription factor *SP1*, but there are 16 shared interactors between the two in BioGRID. *OCT4* and *SOX2* - these two are ubiquitous transcription factors of the basic helix-loop-helix leucine zipper family that form homo- and heterodimers and recognize a CACGTG motif termed E box [7]. Nevertheless, they have no shared interactor in BioGRID. c-*JUN* and c*-MYC* - to the best of our knowledge, no evidence is available between these two transcription factors in human cell specimen. Yet they share 15 common interactors in BioGRID.

These examples indicate that such a model is too simplistic to describe the complexity of TF-TF interactions. They also suggest that while the number of shared interactions might be an informative feature, it cannot be used on its own to correctly separate these three classes.

In this paper, we describe the Network-Augmented Transcriptional Interaction and Coregulation Analyser (NAUTICA). NAUTICA classifies TF-TF interaction predictions produced by a prediction tool like TICA, which considers positional information of binding sites alone, by using the number of shared interactions between the candidate TFs in a PPI network. NAUTICA’s performance is superior to two simpler approaches, viz. using only TICA (or other similar TF-TF interaction prediction tool, e.g. CENTDIST), and using only information coming from the PPI network.

It is worth noting that we use the term “transcription factor” as a generalized terminology. In a strict definition, transcription factors are proteins that control the rate of DNA-to-mRNA transcription by direct binding of DNA through DNA-binding domains. However, here, we include in our set of eligible proteins also those that bind transcription factors on their binding pockets. This generalization makes the problem of deciphering the nature of the interactions of these proteins more interesting (as it includes both interactions on DNA and on binding pockets), as well as more complete (as these proteins are part of the involved transcription machineries.)

## Results

We trained NAUTICA on the training dataset TR (defined in Methods). The thresholds were fitted by maximising recall of each class with respect to TR. A parameter sensitivity analysis was performed to evaluate stability of the measures: results are described in Supplementary Table ST1.1. Optimal values were selected as *τ*_*H*_ = 8 and *τ*_*L*_ = 5. We then applied NAUTICA to each TF pair for which there is a TICA prediction (whether interaction or not) and each TF in the pair has degree at least 3 in BioGRID. There are 32,796 such pairs (and thus 32,796 NAUTICA predictions). Prediction counts are tabulated in Table 1.

**Table 1:** Breakdown of predictions based on class

Class	Count	Percentage
COOP	806	02.46%
COMP	2807	08.56%
NINT	29,183	88.68%
TOTAL	32,796	100.0%

*Note*: Percentages indicate the relative proportion of classes in the output set

### Calibrated confusion matrix shows high levels of recall for NINTs and COOPs

We used dataset TS to evaluate the recall of NAUTICA, subject to the calibrations described in **Recall calibration and evaluation**. The confusion matrices for the fitted parameters both without and with calibrations are reported in Table 2 (upper and lower, respectively). By comparing the two, we observe that, after calibration, the method displays very good recall in predicting co-operations (COOP, 75%) and non-interactions (NINT, 83%). The table also reveals a lower recall for competitions (COMP, 39%), and that there is difficulty in distinguishing between COMP and NINT. On the other hand, if one is interested in distinguishing COOP from COMP and NINT (i.e. one is not interested in distinguishing COMP and NINT), one can expect excellent performance (75% recall for COOP and 98% recall for COMP and NINT combined.)

**Table 2:** Recall estimates on testing set TS

Actual class	P_NINT	P_COMP	P_COOP	Recall
NINT	21	17	13	41%
COMP	3	3	2	37%
COOP	15	2	46	73%
NINT^a^	5292	928	145	83%
COMP^a^	68	49	6	39%
COOP^a^	154	17	520	75%

*Note*: *Upper -* no calibration. *Lower -* with calibration for recall (also marked with ^a^). The calibration is described in **Recall calibration and evaluation** and is performed by substituting to each prediction (whether correct or not) its weight. The weight is defined as the ratio between the number of interactions that have the same N_12 as that prediction and the same count done for the N12 value that has the least interactions. Note that specificity cannot be calibrated without some additional assumptions

### NAUTICA theoretical precision estimation confirms soundness

At the same time, we ran the theoretical estimation described in **Precision estimation** on dataset TS (cf. Methods). Here are the results where we analysed a total of *M* = 32823 pairs. Under the assumption that the proportions of actual NINT:COMP:COOP is 80:10:10, and assuming recalls as in Table 2 (*R*_*NINT*_ = 0.83, *R*_*COMP*_ = 0.39, *R*_*CO*0*P*_ = 0.75), we have an estimated precision of interaction prediction of

$$ {P}_{INT}=\frac{0.040M+0.075M}{0.040M+0.075M+0.136M}=0.46 $$

This estimate of precision is very respectable, given the assumption that there are eight times more NINT than each of COMP and COOP cases in the population; it is circa two folds better than random guessing.

In fact, under a general assumption that the ratio NINT:COMP:COOP is y:x:x (where y + 2x = 1), and the same recalls as before, we can derive P_INT_ = 1.14x/(0.8x + 0.17). Moreover, P_INT_/2x gives the number of folds improvement over random guessing. Thus, when x < 0.14 (i.e. no more than 28% of random TF pairs are COOP or COMP, which seems a safe assumption), the estimated precision is always at least two folds better than random. And this improvement over random monotonically increases as x decreases.

### Comparison with TICA and a PPI-based tree

We compared NAUTICA’s predictions with its two basic components: the TF-TF interaction prediction tool (viz. TICA) and a decision tree based on BioGRID alone. We did this to investigate whether or not the novel method is more effective than its constituents. For what concerns the interaction prediction tool, we ran TICA on TS (an independent test set, described in Section **Recall calibration and evaluation**), and compared the results. Since TICA alone does not distinguish between co-operations and competitions, we combine the two classes into a more general “interaction” class in this comparison. Also, in NAUTICA, we have relaxed our TICA statistical threshold (to 0.3) to increase recall (since NAUTICA is able to filter the corresponding increase in false positives from TICA). The comparison here is done against TICA alone, both using the same statistical threshold (viz., 0.3) and with TICA at its original 0.2 threshold. Results are shown in Table 3. NAUTICA has a 17%-higher recall w.r.t. TICA at the 0.2 threshold when used to predict noninteractions on dataset TS (36%-higher after calibration), with the added capability of being able to distinguish between co-operations and competitions. On these, NAUTICA also exhibits better recall on co-operations (20%-higher after calibration) at comparable recall on competitions. NAUTICA has higher calibrated recall on noninteractions and cooperations, but lower calibrated recall on competitions, than TICA at the 0.3 threshold. However, as noninteracting TF pairs are expected to vastly outnumber interacting pairs in the real population, NAUTICA’s much better recall on noninteractions is a significant advantage that fully compensates for its lower recall on competitions than TICA at the 0.3 threshold in practice.

**Table 3:** Recall estimation versus TICA with respect to testing dataset TS

Actual class	TICA (***p*** = 0.2)			TICA (***p*** = 0.3)			NAUTICA		
	NINT	INT	Recall	NINT	INT	Recall	NINT	INT	Recall
NINT	18	33	35%	18	33	35%	21	30	41%
COMP	4	4	50%	3	5	63%	3	5	63%
COOP	25	38	60%	22	41	65%	38	48	76%
NINT^a^	3868	2497	61%	3868	2497	61%	5259	1106	83%
COMP^a^	69	54	44%	36	87	71%	68	55	44%
COOP^a^	248	443	64%	186	500	73%	154	537	77%

*Note*: COOP and COMP predictions from NAUTICA were collapsed into the general ``interaction” (INT) category for the comparison. *Upper:* no calibration. *Lower:* with calibration (also marked with ^a^). Calibration is done with the same procedure as the general NAUTICA recall analysis (Table 2)

For what concerns BioGRID, we defined a simplified decision tree based only on *N*_*12*_ (i.e., the number of shared interactors in the network, cf. **Methods**), using the following rules. Consider two thresholds *L* and *H* distinct from *τ*_*L*_ and *τ*_*H*_.Then, if a candidate TF pair has a number of shared interactions between 0 and *L* (*L* exclusive), predict non-interaction; if a candidate has a number of shared interactions between *L* and *H* (*L* inclusive, *H* exclusive), predict competition; otherwise, predict co-operation.

*L* and *H* were estimated based on the same training set TR as in NAUTICA, for consistency. The calibration of parameters is shown in Supplementary Table ST1.2; the final thresholds are *L* = 1 and *H* = 10. We ran this simple BioGRID decision tree on TS. The resulting calibrated recall values are *R*_*NINT*_ = 0.79, *R*_*COMP*_ = 0.96, and *R*_*COOP*_ = 0.40; the corresponding theoretical precision is *P*_*INT*_ = 0.45. NAUTICA has better performance after calibration with respect to dataset TS when predicting non-interactions and co-operation (cf. Table 2). While the BioGRID decision tree’s performance on competition looks superior on the surface, it is important to note that it predicts every TF pair that has 1 ≤ *N*_12_ < 10 as a competition. In other words, the BioGRID decision tree ---if we ran it on all candidates, as opposed to just TS---would predict 10,016 out of 32,796 candidates as competitions, or 30.5%, which seems an unrealistic amount. In contrast, among these 10,016 candidates, NAUTICA would predict 6674 as non-interactions, 2760 as competitions and 582 as co-operation. NAUTICA’s categorizations seem more reasonable than the simple BioGRID decision tree. We also note that the BioGRID decision tree’s 4% lower recall on NINT results in a significant number of mispredictions, as NINTs vastly outnumber the other classes.

The NAUTICA decision tree can use any predictor of TF-TF interaction instead of TICA. Candidates are, for instance, CENDIST and TACO. Every predictor has its own limitations. For instance, by using TACO we would selectively focus on co-operations (as TACO predicts dimerization, viz. physical binding, which is most compatible with cooperation), while CENDIST requires both ChIP-seq and a motif database.

### CORUM analysis reveals stronger enrichment for COOPs

Finally, we can use protein complex information to further validate NAUTICA’s predictions. Transcription factors that cooperate to bind DNA as a single unit should have a higher likelihood to be found in protein complex databases. Conversely, competitions and non-interactions should have a low likelihood to be reported as co-complexes. (Competitors bind mutual exclusively to a shared partner to form different complexes. They are thus unlikely---but not completely impossible---to bind each other in a third complex.) Thus, we compare our list of predicted TF-TF interactions to CORUM [8], a curated database of protein complexes. We use the human complex database released on September 3rd, 2018 (available at http://mips.helmholtz-muenchen.de/corum/#download). To estimate the representation of each class in CORUM, we check for each predicted member of the class (COOP, COMP, or NINT) whether there is at least one CORUM complex that contains both constituent TFs. The ratio between this list and the total number of predicted interactions in that class is used to compute the enrichment of that class in CORUM. Note that this is done across the spectrum of predictions available, as opposed to using only the test dataset TS. We report in Table 4 the percentage of COOP, COMP and NINT which are found in CORUM complexes.

**Table 4:** Enrichment of NAUTICA predictions in CORUM

Label	Percentage in CORUM	Fold-increase w.r.t. COOP	Fold-increase w.r.t. COMP	Fold-increase w.r.t. NINT
COOP	26.1%	–	10.88	52.20
COMP	2.4%	0.09	–	4.80
NINT	0.5%	0.02	0.19	–

*Note*: Breakdown by class. Fold-increase is the ratio between the two percentages. In line with the fact that CORUM privileges co-operations, only 2.9% of predicted non-cooperations (COMP+NINT) are supported by CORUM evidence, whereas 26.1% of predicted co-operations (COOP) are supported

The over-representation of COOP cases in CORUM is consistent with and validates our hypothesis. In particular, only 2.9% of our COMP+NINT pairs are in CORUM complexes, while 26.1% of COOP are in these complexes. The slight enrichment in CORUM of competitions over non-interactions is also consistent with the expectation that some competing TFs can be members of the same complex while being mutually exclusive in other complexes. The somewhat low figure of 26.1% of predicted co-operations being found in CORUM is also not unexpected, due to the incompleteness of CORUM.

### Investigation of significant cases shows both novel and known interactions

We manually validated the strongest predictions achieved from our method. We considered the top 40 predictions of co-operations as those with higher N_12_ extracted respectively along the (1) and (0,0) branches of Fig. 4. We then searched these predictions within PubMed articles. We found that, out of the said 40 predicted co-operations, 19 were mentioned in articles, and 12 of them (63%) were mentioned as co-operating or co-binding; out of 40 predicted competitions, 17 were mentioned in articles, and 5 of them (29%) were mentioned as competitions, whereas for many of them a classification based on literature review was not possible. Respectively 21 and 23 pairs out of the above sets were not mentioned in PubMed, they represent original predictions of TF-TF co-operation or competition. The full list is provided in Supplementary Table ST2.

We also investigated three groups of TFs with known biological interactions to evaluate the quality of NAUTICA predictions. First, the triplet comprised of *MAX*, *MYC* and *MNT* is known to engage in competitive behaviour. Specifically, *MYC* is competing with *MNT* to bind to *MAX* and form a heterodimer. NAUTICA correctly predicts both the *MAX*/*MYC* and *MAX*/*MNT* (BioGRID and TICA predict interaction) co-operative behaviour, while the *MNT*/*MYC* pair is predicted as competitive due to the lack of shared BioGRID edges. This behaviour is confirmed by several experimental studies. Similar results can be obtained by substituting *SIN3A* to *MNT* [9] [10].

Consider now the cohesin subcomplex *RAD21* / *SMC1* / *SMC3*. Cohesin is involved in DNA looping [11]. NAUTICA correctly predicts the co-operation of *SMC3* and *RAD21*, while predicting the competition of *HDAC2* with *SMC3*. Since *HDAC2* is involved in the chromatin compacting processes caused by DNA deacetylation, it is reasonable that it competes for the same binding spots as cohesin; *RAD21* and *HDAC2* are predicted to have no interaction, which makes sense because *RAD21* acts as a bridge between the *SMC* subunits of cohesin and bears little direct effect on the DNA binding of that complex [12].

Finally, evidence has been found of a competitive behaviour between Early Growth Response 1 (*EGR1*) and the TATA Box-binding Protein *TBP* [13]. Although NAUTICA predicts no interaction between the two (due to lack of predicted TF interaction), it does predict a competition between *EGR1* and the *TBP*-Associated Factor 1 (*TAF1*), which is required for the formation of the *TFIID* complex containing *TBP* [14]. Thus, it is possible to hypothesize that *EGR1* is in fact competing for the binding spots of *TAF1*, and preventing the recruitment of the same for the formation of the *TFIID* complex, resulting in an apparent competition between the two.

## Discussion

In this paper we have presented NAUTICA, a novel methodology that improves upon our TICA framework (and other similar framework based on TF binding-position information), and aims at enriching the previous model by classifying predicted interactions as co-operations or competitions. It also corrects previous examples of false positives, by eliminating non-interacting TF pairs which were reported by ChIP-Seq to bind in the same promoters; this might be because the ChIP-Seq experiments were conducted on individual TFs separately and hence peaks located on near-by positions might not be on the same instances. Although we performed all validations using TICA as a reference framework for TF-TF interaction, the NAUTICA model can be used with other similar frameworks based on TF binding-position information. Examples of these are TACO and CENTDIST. We did not incorporate these into NAUTICA as we do not own these tools.

To the best of our knowledge there is no method that performs wide-ranging TF interaction classification, so NAUTICA is a new contribution to the field. Several methods perform predictions on TF-TF cooperation, such as [15], but these methods require TF binding motif predictions and/or knockdown experiments, making comparison difficult.

NAUTICA shows good levels of recall with respect to all different interaction classes, especially after calibration with respect to the density of each *N*_*12*_ bin, making it a powerful tool for TF-TF interaction classification. It works well in separating co-operating from competing TFs (cf. Table 2), which is of interest since (to the best of our knowledge) there is no other computational method that makes the same distinction. The enrichment of co-operation predictions with respect to CORUM complexes is consistent with biological intuition, further supporting our claim that NAUTICA can correctly distinguish between co-operations and competitions. Our estimation of precision, while penalized by the scarcity of known TF-TF competition cases in the literature, is still significant at 45%, which is some two-folds better than random guessing under the assumption that the ratio NINT:COOP:COMP is 8:1:1. Moreover, the smaller the faction of interacting TF pairs is among all possible TF pairs in real life, the better this improvement over random is.

Even when one is not interested in the distinction between co-operating and competing TFs, NAUTICA predictions are still very useful. Table 2 reveals that noninteracting TF pairs which are mis-predicted by TICA as interacting are about 6 folds (= 928/145) more likely to be mis-predicted as competing than as co-operating pairs. Hence large fractions of false-positive interacting TF pairs from TICA predictions can be eliminated by dropping those that NAUTICA classifies as noninteracting or competing.

The choice of parameters used for NAUTICA is supported by the distribution of the number of shared interactions *N*_*12*_ across the various bins we defined, which marks an over-representation of co-operating TF-TF pairs for high values of *N*_*12*_ and conversely an under-representation at smaller *N*_*12*_. The relatively high count of co-operating TF-TF pairs at *N*_12_ = 0 (and other very small *N*_*12*_ values) is likely due to incompleteness of the PPI network. The results are nonetheless consistent with our model assumptions and indicate that NAUTICA is using sensible parameters in its decision points. Bins 0, 8, 9 and 10+ are highly significant, providing further evidence to our claims. We note that bins 1, 2, 3, 4 and 5 are not significant according to the *χ*^2^ test, indicating that the co-operation claim in those bins is harder to support (though the existence of direct PPI edges in BioGRID and positive TICA predictions help resolve cases in these bins). Details on the *χ*^2^ test and the relative risk/odds ratio analysis of *N*_*12*_ bins are given in Supplementary File 1.

Validation for NAUTICA classification is easily done with respect to the co-operation class, for which literature is more readily available, but trickier for the competition cases. It is indeed harder to find direct competition evidence in the literature. However, by using indirect evidence such as CORUM, we show that NAUTICA has solid biological premises and distinguishes the competitive and co-operative cases.

There is an interaction type that can be classified as between COMP and NINT, and is worth discussing in further detail. Let *X* and *Y* be two competing TFs and let *Z* be a third TF such that *X* recruits *Z* and *Y* does not recruit *Z*. Assume that *Z* is unlikely to bind certain promoters without recruitment by *X*. Thus, when *X* binds those promoters, *Z* also binds; and when *Y* binds those promoters, *Z* does not bind. In this case, strictly speaking, (*Y*, *Z*) is not a COMP interaction by our definition of COMP; yet (*Y*, *Z*) may have characteristics similar to genuine COMP pairs. In particular, (*Y*, *Z*) is likely to bind to the same (or close-by) spots in a mutually exclusive manner. Yet experimentally, *Z* cannot be shown to block *Y* (i.e., over-expressing *Z* does not prevent *Y* from binding promoters.) An example of this dynamics is *HDAC1*/*TBP* where *AP4* competes with *TBP* and *AP4* recruits *HDAC1* [16]. Based on this, we can divide the COMP class into two mutually exclusive subclasses: COMP+, which are supported by TICA and likely compete for the same binding spots; and COMP-, TFs that are members of different complexes that in turn compete for binding spots in DNA, though the two TFs do not compete directly for the same binding spots. We hypothesize that this last group can be found in the bottom left branch of Fig. 4, with *HDAC1*/*TBP* corroborating this idea, but further investigation will be required.

Some caveats have to highlighted however. Our use of the term “transcription factor” is more lax than the classical definition, as mentioned in **Background**. The same can be said for our use of the terms “cooperation” and “competition” among TFs, since we include interactions happening in the binding scaffold(s) of transcription factors. Moreover, some TFs may co-operate and compete in different contexts from the ones mentioned above, but the current model is not suitable to predict these cases. It should also be noted that the NAUTICA decision tree is based on data from BioGRID, but BioGRID data originated from articles which refer to different conditions and organisms. Moreover, both BioGRID and CORUM might be biased towards well-studied proteins. Finally, the NINT:COOP:COMP split that we use in providing precision estimates is just an educated guess; there is no compiled resource that reports the real ratio of COOP, COMP, and NINT in cells.

## Conclusions

We propose NAUTICA, an improved version of our existing TICA framework. It leverages protein-protein interaction network information (specifically, the number of shared interactors between two TFs in the network) for further classifying current TF-TF interaction predictions into co-operations and competitions. NAUTICA predictions are supported by both existing protein-complex databases, literature validation, and improve on the performance of TICA. NAUTICA is a novel, effective tool for interaction classification that does not require motif prediction, and is robust with respect to the incompleteness of the reference PPI network (in our case, BioGRID).

## Methods

### Conceptual description

To address the problem of TF-TF interaction classification, we propose using information contained in PPI interaction networks to augment the already significant discerning power of TICA (although other TF interaction prediction tools based on binding site information could be used as well in its place). NAUTICA is based on the following considerations: TF-TF co-operation usually [17] (although not always) entails one of the two interactors recruiting its cognate partners to the same binding location, whether because the binding of the first is a catalyst of the second or because they bind DNA as a single macromolecule. Therefore, if two transcription factors are cooperating, they tend to be a part of the same transcriptional complex; also, these complexes tend to be large and composed of several subunits working together [18]. Therefore, two co-operating TFs are likely to share quite a few common interactions in a PPI network and are likely observed to have direct interaction in a PPI network.

In contrast, TFs that compete for a shared partner generally attempt and bind a transactivation domain on the target partner, most often to the exclusion of each other. Similarly, two TFs that compete for the same site on the promoter of a target gene also exclude each other [19]. This means that they are unlikely to directly bind each other. Furthermore, as a consequence of the previous point, factors that compete for the same partner or site tend not to share many common interactions in a PPI network (since they are unlikely to belong to the same complexes). On the other hand, if two TFs share a high number of common interactions in a PPI network and yet are not observed to have a direct interaction in the PPI network, a possible explanation is that they are competing for these shared interactions.

Yet, the number of shared interactions in a PPI network is a not a clear predictor of the nature of the interaction. This due to two reasons: first, human PPI networks are incomplete [20]; second, the more interactions one of the two TFs has, the more likely it is to share some partners with any other TF due to sheer coincidence. Moreover, it is also difficult to distinguish competitive TF-TF interactions from non-interacting pairs of TFs based on the number of shared interactions in a PPI network alone, since both kinds of TF pairs are likely to have a low number of shared interactions.

While the first and second considerations above can be tackled using PPI network information alone, the third one by definition requires other information to compensate for the former’s deficiencies.

### Protein-protein interaction network

Our reference PPI network for this work is BioGRID [5], a resource which organizes and archives genetic and protein interaction data from several model organisms (including humans). We use the database consisting of all human interactions, version 3.4.162, available at http://thebiogrid.org/download.php. We only consider physical and multi-validated (cf. https://wiki.thebiogrid.org/doku.php/biogrid_mv) interactions in BioGRID. The resulting network contains 8590 human proteins connected by 34,907 edges. Of these proteins, 763 are known human transcription factors.

We restrict our attention to nodes that are TFs and have degree at least equal to 3 in the full network. This is done is order to filter out all those TFs that are isolated due to the incompleteness of the network, and to remove any disconnected 2-node islands that are likely to be 1-to-1 binding without relation to the other proteins, or are in regions of the PPI network that are likely more incomplete; also, TFs with too few edges cannot have a significant number of shared edges with their neighbours, limiting the effectiveness of this feature (see below).

**Fig. 1:**
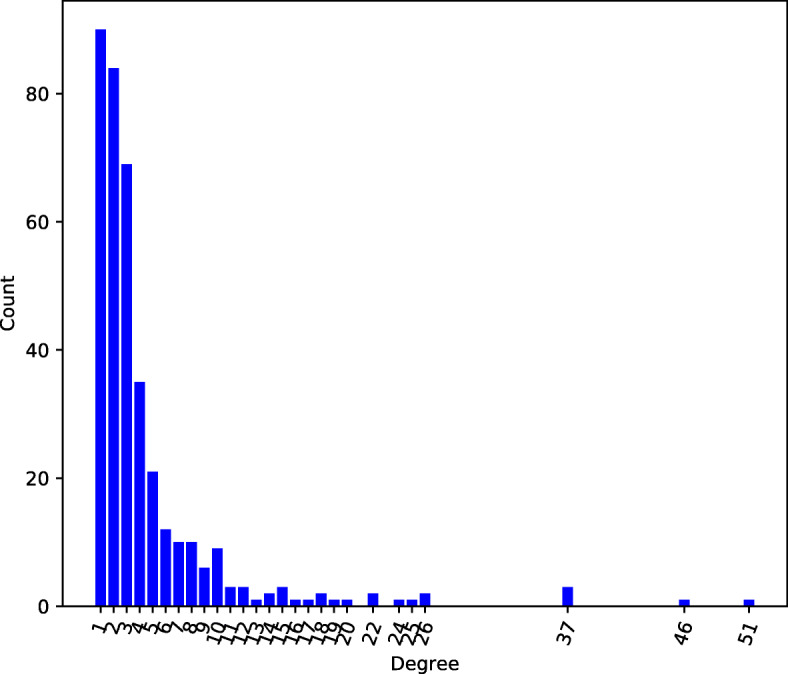
Degree distribution of TF-TF interactions in the human, physical, multi-validated BioGRID network. The distribution demonstrates a power law-like shape. Note that we eliminate from the complete network (viz., including non-TF proteins) those nodes with less than 3 interactions; however, in this graph restricted to TF-TF edges only, fewer (1, 2) interactions are possible

After filtering, we are left with 375 human transcription factors, having an average degree of 4.5. In Fig. 1 we show the distribution of the number of TF-TF interactions in the network, viz. the number of TF-TF only edges in the filtered network consisting entirely of these TFs and their interactions. The degree distribution exhibits a power law-like shape, which is typical of scale-free networks [21].

**Fig. 2:**
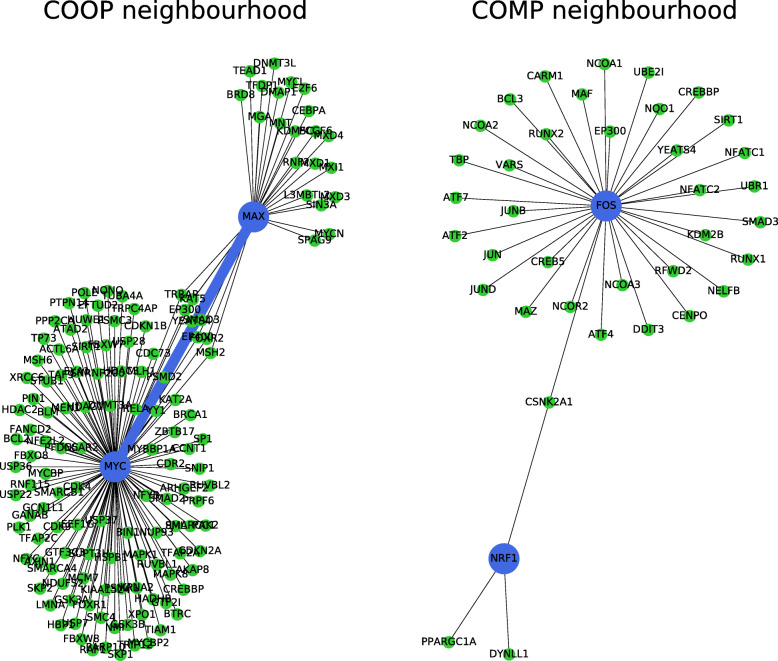
Comparison of the neighbourhood of *MAX* and *MYC* with that of *FOS* and *NRF1* in BioGRID. Blue line denotes direct connection in BioGRID between *MAX* and MYC. Note the batch of shared interactions between *MAX* and *MYC* (left) as opposed to the single CSNK2A1 being shared by *FOS* and *NRF1* (right)

Let *N*_*12*_ be the number of shared interactions between two proteins in the PPI network. To visualise the nature of this measure, imagine that two proteins are two colleagues in the same work network and an edge exists between them if they collaborated in at least one project. Then *N*_*12*_ can be thought of as the number of shared co-workers that two colleagues have. The higher this number, the more likely is that the two are working on the same project and/or they share common interests. This approach is reminiscent of co-citations used in link analysis [22]. In Fig. 2 we compare the shared neighbours of two prominent TF pairs: *MAX*/*MYC* (left), a known dimerising pair, and *FOS*/*NRF1*, a competing one.

**Fig. 3:**
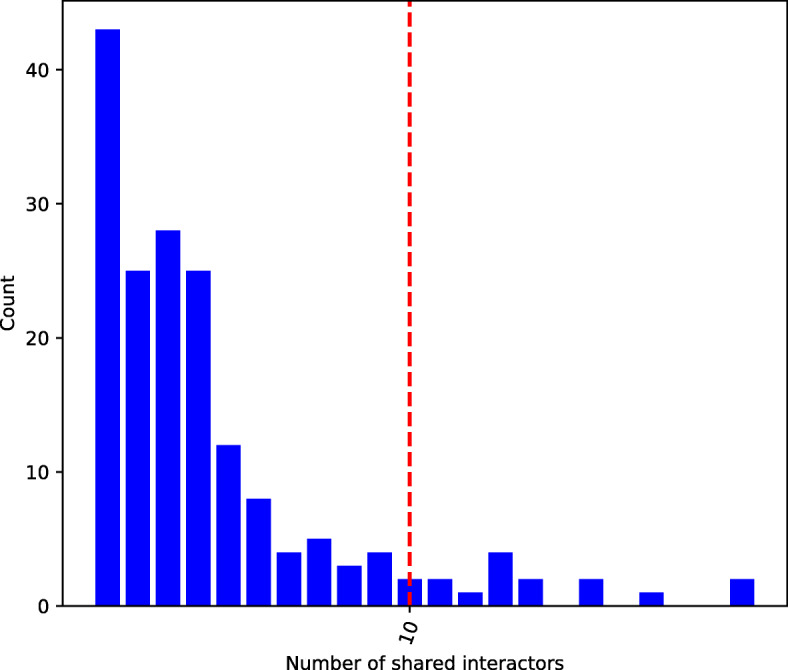
Distribution of the number of shared interactions between TFs in the human, physical, multi-validated BioGRID network. The number of shared interactions between two TFs is denoted *N*_*12*_. The red line denotes *N*_*12*_ = 10, which we consider the beginning of the distribution tail

We show in Fig. 3 the distribution of *N*_*12*_ across TFs in the filtered BioGRID network; it also exhibits a power law-like shape. Note that *N*_*12*_ is computed considering edges connecting TFs to both TF and non-TF proteins in the general network, since TFs can sometimes interact with non-TF proteins [23].

Observing the distribution in Fig. 3, we notice that for *N*_12_ ≥ 10 there is a drop in the number of edges having high values of *N*_12_. As there is no standard way to define the beginning of a distribution’s right tail, we define the tail of the distribution (viz., the portion of the distribution which is significantly different from the rest) to start at *N*_*12*_ = 10. Thus, we collapse the tail and split the PPI shared-interactor distribution into eleven bins: *N*_*12*_ = 0, *N*_*12*_ = 1, …, *N*_*12*_ = 9, and *N*_*12*_ = 10 + .

### TF-TF interaction prediction

In our previous work [2] we presented TICA, a statistical algorithm for predicting whether two transcription factors interact based on positional information from ChIP-Seq experiments. We use TICA here as a source of TF-TF interaction candidates for NAUTICA; any other TF-TF interaction prediction tool can be used for this purpose.

TICA is based on the concept of minimal distance couple. Briefly, binding sites from ENCODE narrowPeak datasets are reduced to their 1 bp point source, and their positioning is compared across promoters of active genes. Whenever two binding sites of two candidate interactions are found to be the closest to each other and below a significance threshold, they are paired and form a minimal distance couple (or mindist couple). Each mindist couple defines a mindist couple distance (distance between the two closest ends), and all mindist couples generate a distance distribution that is associated to the two candidates. This distribution is used to infer interaction by using statistical tests: its median, average, median absolute deviation and right tail size are compared to those of the null distribution for the context cell lines. If at least 3 of these measures are significantly closer to 0 with respect to the null distribution, the TF pair is predicted to be interacting.

In the context of NAUTICA, TICA is used with the following parameters: *P* value 0.3 on all four tests, of which at least 3 are required to call a prediction, based on null distributions that must have at least 1% mindist couples located in promoters.

### NAUTICA classification rules

Neither a pure PPI network analysis nor the TICA framework (or other binding-site position-based frameworks) provide enough evidence for a clean-cut classification of TF-TF interactions. We present the NAUTICA set of decision rules to tackle this task.

### Class nomenclature

A pair of TFs which is submitted for classification is called an *interaction candidate pair*, and the two TFs in the pair are called *interaction candidates*. An interaction candidate pair can be predicted as one of three classes. *Co-operations* (COOP) identify TFs that bind the DNA as a single macromolecule, or those where one interactor binds the DNA first and then recruits the other for binding; *competitions* (COMP) identify TFs that compete for the same binding spots in the DNA, or that compete to bind in a mutually exclusive way to the same partner and subsequently bind the DNA in the same (or close) spots; and finally, *non-interactions* (NINT) are TF pairs do not interact with each other in any physical way - they neither compete nor attempt to bind with each other to form complexes.

An interaction candidate pair which is either predicted as COOP or COMP is referred more in general as an *interaction* prediction. This is useful when comparing NAUTICA with tools that predict TF-TF interactions but not the nature of interactions on the same test set(s).

### Decision tree explanation

**Fig. 4:**
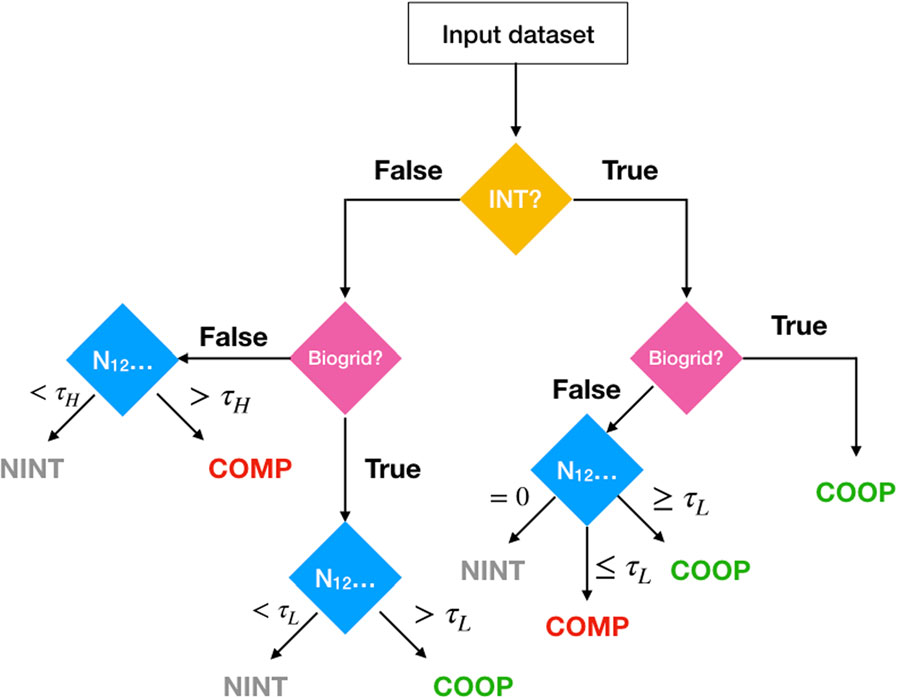
NAUTICA’s decision tree. Input data consists of interaction prediction labels, BioGRID edge extraction and number of shared interactions for a give TF interaction candidate. τ_L_ is the threshold on *N*_*12*_ that separates NINT from COOP predictions for interactions supported by BioGRID only, and similarly separates COOP and COMP predictions in pairs supported by the interactions predictor only. τ_H_ is a different *N*_*12*_ threshold that separates NINT from COMP in the event that no direct interaction evidence is found

NAUTICA’s set of decision rules is summarized by the decision tree in Fig. 4. NAUTICA’s decision tree is the result of several attempts at feature definition and quality measure estimation. Among others, different functions of the number of shared interactions *N*_*12*_ have been studied, such as the CD distance [24] and the Jaccard Index of shared interactions (which we define as the number of shared interactions between two candidate TFs divided by the union of all interactors of the same). We evaluated the separation power of the features by using the reference training dataset (cf. **Model Training**). However, the current decision tree proved to be the best so far, with the additional benefit of simplicity, as we show in the following.

The three main components of these decision rules are the interaction prediction value, a Boolean value equal to 1 if and only if a direct interaction is predicted by the supporting interaction prediction tool – for this study, we use TICA predictions in any of three available cell lines, HepG2, GM12878 and K562 (multiple cell lines do not offer additional support); the existence of a BioGRID direct edge, a Boolean value equal to 1 if and only if a direct edge is found in BioGRID between the two interaction candidates; and the number of shared interactions *N*_*12*_. Recall that an interacting protein is shared between two TFs if there is a direct edge between said protein and both members of the candidate pair.

These features are proposed based on the following reasoning: TICA predicts physically interacting factors with high reliability. The number of shared interactions in the PPI network parameterizes the size and number of putative common regulatory modules they belong to; a large number of shared interactions suggests being in the same complex and thus co-operation, whereas a small number of shared interactions suggests the opposite. Adding in BioGRID direct edges augments the recall of the model, accounting for any interactions which evade detection by binding-site location analysis by TICA; and also provides evidence of being in the same complex, a sign of co-operation.

### Model training

As shown in Fig. 4, NAUTICA considers three decision features; viz. the interaction prediction by TICA (or in principle, any tool), the direct edge in BioGRID, plus a fitted two-tiered thresholding τ_L_ and τ_H_ on the number of shared interactions in BioGRID, *N*_*12*_.

To fit the two thresholds τ_L_ and τ_H_ in NAUTICA, we curated an initial training set (denoted TR) of 110 TF interactions by sampling the list of possible TF pairs for which interaction prediction data is available. The sampling was done by randomly choosing groups of 10 TF pairs, each having a number of shared interactions *N*_*12*_ belonging to a different bin described in **Protein-protein interaction network**. Each sampled pair was labelled (as COOP, COMP, or NINT) by manually checking current literature. Too few of these turned out to be competition interactions, so we fleshed out the list with additional TF pairs mentioned in the papers that we read while doing the manual checking above, and curated the nature of their interaction as well. We also ensured that the set of TF pairs sampled from each bin has equal representation of pairs having direct PPI edge and pairs having no direct PPI edge. The complete training set consists of 175 labelled TF-TF interactions (full list provided in Supplementary Table ST3.1). Out of these 175, 28 were found to be competitions, 112 as co-operations and 32 as non-interactions. Three interactions (*JUN* / *JUND*, *EGR1* / *SP1* and *RELA* / *SP1*) could be classified into multiple categories based on available evidence, and thus were excluded from the threshold-fitting process. This proportion of co-operations to competitions to non-interactions is not representative of the expected distribution of such interactions and non-interactions in vivo; thus, we implemented a calibration system to better estimate the quality of our predictions in light of the relative density of shared interactions (cf. below).

Of the two different values fitted, τ_L_ is used to distinguish between co-operation and competition in the case of interaction evidence while τ_H_ is instead used in the case where no interaction is predicted by TICA and/or BioGRID. Some leeway has been given to the thresholds in order to avoid overfitting to TR.

### Recall calibration and evaluation

To test the set of NAUTICA decision rules, a different set of TF interactions is curated, denoted TS.

TS is used to evaluate the recall of NAUTICA. We curated a separate list of 164 test cases, by sampling uniformly across the different values of *N*_*12*_ (using the same procedure described in Section **Model training**, above; the full list is provided in Supplementary Table ST3.2). No member of this test set is shared with TR. For each test case in TS, we investigated the literature for evidence of interaction and the nature of it; this resulted in 13 competitions, 95 co-operations, and 55 non-interactions. We do not, however, have ChIP-Seq data available for some of these curated examples (which are required for TICA predictions in NAUTICA). Thus, TS contains 51 non-interactions, 8 competitions and 64 co-operations - those for which data is available.

NINT (non-interactions) cases are expected to be the majority of predictions [25], but they are also the most difficult to validate due to lack of experimental reports on them. As such, a face-value evaluation of the recall on the NINT (as well all other classes) would be strongly misleading; to solve this problem, we implemented a calibration system to estimate the number of correct/incorrect prediction based on an expected distribution of each class, details as follows.

**Fig. 5:**
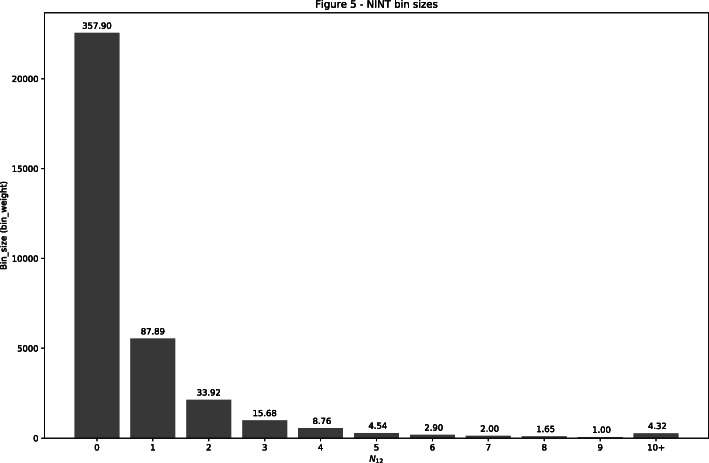
Binned NINT null distribution of ***N***_***12***_**.** The NINT null distribution of the number of shared interactions in BioGRID between TFs for which TICA has information for analysis. On each bar, we report the relative bin weight computed with respect to bin 9 (the smallest)

Figure 5 shows the binned distribution of *N*_*12*_ in BioGRID for each TF pair for which TICA has data available for analysis and satisfying the filters in **Protein-protein interaction network**: this is done in order to not confound the distribution with interaction groups where many pairs are not available for predictions. We call this distribution the NINT null distribution, because most of the TF pairs spanned by this distribution are expected to be non-interacting pairs.

We use this distribution to derive the relative weight of TF pairs curated as NINT when used for recall evaluation as follows. Let the bin that contains the least number of candidates be the “base bin”, and assign it a weight of 1. Each of the other bins is assigned a weight which is its population size divided by the population size of the base bin, rounded down to the closest integer. Based on the complete distribution in Fig. 5, the base bin is bin 9. Then each TF pair curated as NINT is weighted according to the weight of the bin it is in. For example, a TF pair curated as NINT that has 0 shared interactor in BioGRID (and thus is in bin 0) is given the weight of 358. Consequently, if this TF pair is correctly predicted as NINT, this counts as 358 correct predictions; on the other hand, if it is incorrectly predicted as anything else, this counts as 358 wrong predictions.

There are too few COMP cases to form a null distribution of their own, and it is very difficult to find clearly documented cases of direct TF-TF competition in literature. We have already observed (cf. **Conceptual description**) that competing TFs tend to not be part of the same modules, due to having opposite effects. It follows that in general, proteins that exhibit competitive behaviour should have fewer (if any) shared interactors than those that exhibit cooperative behaviour. Thus their *N*_*12*_ distribution should be closer to the one of NINT pairs. So we also use the NINT null distribution to weight TF pairs curated as COMP interactions.

**Fig. 6:**
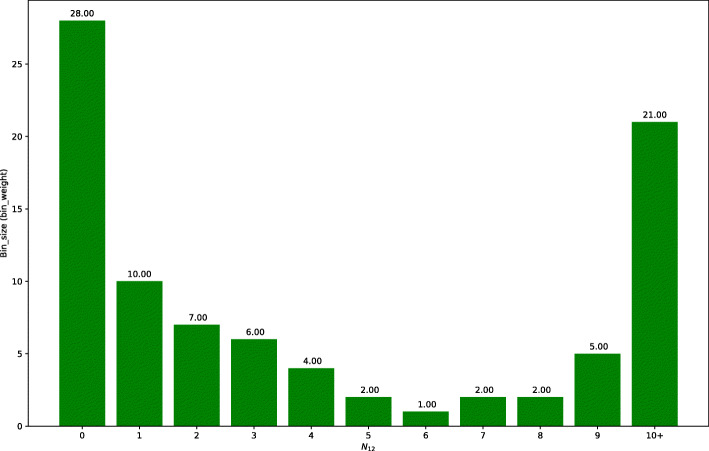
Binned COOP null distribution of *N*_*12*_. The COOP null distribution of shared interactions between cooperating TFs from the training set, restricted to TFs studied by TICA. On each bar, we report the relative bin weight computed with respect to bin 6 and 7 (tied for smallest)

As for the COOP null distribution, 86 out of 112 co-operation cases in TR have TICA datasets available; this is good enough for a representative COOP null distribution (presented in Fig. 6). Bins 6 is the smallest for the purpose of weights. As we suspected, there is a large difference from the NINT null distribution with regards to both distribution shape and weights for all bins, which confirms the necessity of a different null distribution for calibrating the weight of TF pairs curated as COOP interactions.

These distributions are useful in correctly estimating the sensitivity of NAUTICA. Recall that sensitivity for each class is defined by the number of edges correctly predicted to it divided by the total number of edges having that class in the database. However, it is not sound to consider each edge as having the same weight. This is because, due to chemical and physical constraint, two random proteins are more likely to not have any shared edges than the opposite. Moreover, recall that our training dataset is obtained from a uniform sampling of each N_12_ bin, whereas the real population size of N_12_ bins is not uniform (the population size of these bins are well known to follow power law-like distributions.) So our training dataset does not reflect the underlying distribution of N_*12*_ across all different proteins. Thus, if we assume the underlying distributions of N_*12*_ shown in Figs. 5 and 6 are roughly consistent with what can be found in nature, which they seem to be, then we can expect each correct prediction to correspond to *n* correct predictions in a more complete database that is fidel to the real population size distribution of N_12_ bins, where *n* is the relative size of the bin with the respect to the smallest (assumed to be 1, as it is the closest to what we have in our training set). Recall can then be estimated in a calibrated manner. The weight of a TF pair (in the dataset TS) that has *n* shared interactions is calibrated as follows: if its curated label is COOP, then its weight is the weight of bin *n* in the COOP null distribution; analogously, if its curated label is COMP or NINT, then its weight is the weight of bin *n* in the NINT null distribution. Say the assigned weight of a TF pair is *m* (function of its bin). Then NAUTICA’s prediction on this TF pair is counted as *m* predictions. Thus, if this prediction is correct, it is counted as *m* correct predictions; and if it is wrong, it is counted as *m* wrong predictions. For example, let’s assume that a curated non-interaction is in fact predicted as cooperation and has 0 shared interactor in BioGRID. Based on the NINT null distribution shown in Fig. 5, we would add 358 predictions to the confusion matrix entry matching “actual NINT, predicted COOP” (which, incidentally, is a false negative for the NINT class and a false positive for the COOP class).

### Precision evaluation

There are some subtleties that have to be considered when comparing precision values between different predictors. The weights calibration derived from the binning on the number of shared interactions in BioGRID (cf. above) are class specific, i.e. they say nothing about how many more times COOP or COMP pairs there are in any given bin with respect to NINT pairs. Since precision is a measure based on two classes (e.g. COOP vs non-COOP), it cannot be directly applied if the two classes have different weight calibration.

To tackle this issue, we perform a theoretical estimate of precision from TS using the calibrated recall, based on some additional assumptions. Let *M* be the total number of test candidates to be analysed (in our case, this means those TF-TF pairs that have ChIP-Seq data for TICA analysis and where both members have at least 3 interactions in BioGRID). Suppose an 80/20 split between non-interacting and interacting TF-TF pairs, and a further 50/50 split of interacting pairs in co-operations and competitions, for a final 80/10/10 split. This means that we have 0.8 · *M* non-interacting pairs, 0.1 · *M* cooperating pairs and 0.1 · *M* competing pairs. Given calibrated recalls *R*_*i*_ (*i* being any of the three classes, NINT, COOP or COMP), the total number of non-interactions correctly predicted can be estimated as 0.8 · *M* · *R*_*NINT*_ and thus the number of mispredicted non-interactions is 0.8 · *M* · (1 − *R*_*NINT*_). Likewise, 0.1 · *M* · (1 − *R*_*COOP*_) co-operations and 0.1 · *M* · (1 − *R*_*COMP*_) competitions are correctly predicted as such. Thus we can estimate the precision (defined as the number of true positive per predicted positive, $\frac{TP}{TP+ FP}$) of interaction (either co-operations or competitions) as

$$ {P}_{INT}=\frac{0.1\cdotp M\cdotp {R}_{COOP}+0.1\cdotp M\cdotp {R}_{COMP}}{0.1\cdotp M\cdotp {R}_{COOP}+0.1\cdotp M\cdotp {R}_{COMP}+0.8\cdotp M\cdotp \left(1-{R}_{NINT}\right)}. $$

The NINT:COOP:COMP split of 80:10:10 in the formula above is just an educated guess. Precision under other reasonable splits of NINT:COMP:COOP can be calculated analogously.

## Reviewer comments

1) Zoltán Hegedüs

Reviewer Recommendation Term: Endorse publication

Quality of written English: Acceptable

Reviewer summary

This manuscript describes an algorithm called NAUTICA (Network-Augmented Transcriptional Interaction and Coregulation Analyser) that was primarily designed to work as a supplement of TICA software. TICA (Transcriptional Interaction and Coregulation Analyzer) is a previous development of the same lab, performs ChIP-seq based predictions concerning the physical interactions of transcription factors (TF). While TICA makes predictions only about the existence of TF-TF interactions NAUTICA was designed to further refine these results and classify the interactions based on their predicted competitive or co-operational behaviour. NAUTICA predictions are based on protein-protein interaction data derived from BioGRID database. The number of the shared protein interaction partners of the investigated TF pair members are used in a decision tree classification algorithm. Investigations made on manually collected literature based data sets (Fig5 and 6) clearly demonstrates that the number of shared neighbours indeed correlates with the cooperating behaviour of TF pairs, so applying them in functional predictions might be a progressive innovation.

*RESP: We are grateful for the correct description and positive evaluation.*

Reviewer recommendations to authors

Major comments

1)------------- The manuscript describes NAUTICA algorithm but no software implementation of the algorithm available, so there is no direct way to check or reproduce the published results. TICA web service is also unavailable under http://geco.deib.polimi.it/tica/. When I tried to access it I always got "502 Bad Gateway" error message.

*RESP: TICA is a Web application and it relies on a server, which was offline for long time during space relocations in our department at Politecnico di Milano. It is now available.*

2)------------- In NINT class NAUTICA could produce better classification efficiency if the decision tree output was "calibrated" according to the relative size of the N12 bins (Table 2). However, it was not clarified in the manuscript what theoretical assumptions led to the choice of this approach. It should be explained why one prediction event is more important (get more weight after "calibration") than another in the sensitivity (recall) calculation that will finally produce an overall measure of prediction efficiency. If the different N12 bins considered to have some special characteristics affecting the prediction efficiency of the decision tree, separate sensitivity (recall) values could be calculated for each of them reflecting the success rate of the algorithm in that particular bin.

*RESP: We agree on the need of commenting on our calibration; thus we added an explanation of the need for calibration. Firstly, two random proteins cannot be assumed to have equal probability of having/not having a connecting edge. Secondly, the population size of different N*_*12*_ *bins is observed to be rather different. Thirdly, the proportion of COOP/COMP/NINT is rather different in different N*_*12*_ *bins. Due to practical limitations, we are unable to collect a separate test set that faithfully follows these observed population distributions. Instead, we calibrate/weight each test sample so that the calibrated/weighted test set follows the observed population distributions. We have now explained this in more details in “Methods – Recall calibration and evaluation” Section).*

Ensuring a test set’s fidelity to actual population distributions is an important methodological point that is often overlooked. If a test set is not faithful to actual population distributions, accuracy/precision/etc. performance measures determined from the test set may considerably deviate on real data. For example, let’s say we have a well-trained classifier C with 90% sensitivity and 90% specificity. On a test set A where the positive samples fully reflect the properties of positive samples and negative samples fully reflect the properties of negative samples but the proportion of positive to negative is 100:1000, the precision of this classifier C on test set A will be 90/(90+100) = 47%. On a test set B where the positive samples fully reflect the properties of positive samples and negative samples fully reflect the properties of negative samples but the proportion of positive to negative is 1000:100, the precision of this classifier C on test set B will be 900/(900+10) = 99%. As you can see, the performance changes dramatically on different class proportion. If the actual population distribution is 1000:100, the performance on test set B is one which will give us a better sense of what the performance of classifier C will be on real data, whereas the performance on test set A will completely mislead us. Given an actual 1000:100 population distribution, test set A can be calibrated, so that every positive sample counts as 100, while every negative sample counts as 1 post this calibration, the “proportion” of positive to negative in test A becomes 100*100:1000 (= 10000:1000 = 1000:100), faithful to the actual population, and the calibrated precision becomes 99%, more closely reflecting the performance of classifier C that one can expect on real data (assuming the positive and negative samples in test set A are indeed respectively representative of the positive and negative populations.)

3)---(p7 l25)--- "Also note that in NAUTICA we have relaxed our TICA statistical threshold (to 0.3) to increase recall (since NAUTICA is able to filter the corresponding increase in false positives from TICA): the comparison here is done against TICA alone with its default statistical threshold (which is 0.2)." The added value of NAUTICA approach could exactly be evaluated only if TICA is used with the same statistical threshold in both case.

*RESP: When people use TICA, they will use TICA with its standard/recommended threshold. Hence it is appropriate to compare NAUTICA to standard TICA. We thus should provide results comparing to standard TICA. However to clarify this doubt (which is reasonable) from the reviewer, we provide also a comparison of NAUTICA to TICA with the more relaxed 0.3 threshold. Of note is the fact that using TICA alone with this 0.3 threshold significantly lowers its performance in practice when compared to NAUTICA and to TICA alone with its standard 0.2 threshold, as at the lax 0.3 threshold TICA misclassifies many more NINTs as COMPs and COOPs (Data not shown).*

4) ---(p8 l1)--- "Table 3. NAUTICA has double the recall of TICA when used to predict noninteractions on dataset TS," In Table 3. the noninteracting recall data are as follows: no calibration TICA 35% NAUTICA 41% with calibration TICA 61% NAUTICA 83% Both are far from doubling in TICA-NAUTICA relation.

*RESP: This section was unclear. We intended to say that calibration gives to TICA and to NAUTICA, independently considered, about a doubling of recall. I.e. both TICA and NAUTICA’s recall on NINT become doubled post calibration. We have rewritten the paragraph describing Table* *3**(see: ”Results - Comparison with…” Section)*

5) ---(p8 l20)--- "The calibration of parameters is shown in Supplementary Table ST1.2;" There is no Table ST1.2 in the submitted manuscript, however the SM1.1 and SM1.2 tables which aren't referenced in the text at all, contains similar information. This sentence is about the calibration of L and H parameters where the SM1.2 table should be referenced. The calibration procedure of TH and TL parameters (SM1.1 table) isn't mentioned in the manuscript at all. Authors should also discuss why the TH = 8 and TL = 5 and the H = 8 and L = 1 parameter thresholds were finally chosen for the classification procedures. Based on the SM1.1 and SM1.2 tables it also appears that only part of the possible parameter combinations were tested (e.g. L(1-8) with H(8) and L(1) with H(5-11), but a comprehensive test of all possible parameter pair values would be necessary for the unambiguous determination of the optimal classification thresholds.

*RESP: We corrected table references, and Table ST1.1 is now referenced in the text. Regarding complete calibration, we acknowledge the reviewer’s point. At the time of submission we did run a more comprehensive analysis of the L-H parameter space. However, selecting a parameter based on maximizing recall and/or precision is not straightforward. Indeed, H and L are by nature of the model in opposition to each in terms of prediction capability of different classes. In other words, by increase recall in say, COOP, one must sacrifice recall in NINT. In this situation, selecting the optimal threshold proved impossible. We chose instead the numbers 8,5 which have an acceptable recall among all classes. In parameter S1.1 we report a subsample of this analysis, to illustrate how this phenomenon pans out.*

6)---(p11 l31)--- "In this paper we present NAUTICA, a novel methodology that improves upon our TICA framework (and other similar framework based on TF binding-position information)," The statement in the brackets wasn't investigated and proved in the manuscript. It is mentioned in other part of the manuscript that NAUTICA can be used with different preliminary interaction predictors like CENDIST and TACO, however the decision tree parameters were optimized only for input originated from TICA, so there is no information about its efficiency with other upstream software.

*RESP: The observation is correct; we clarified in the text that we did our comparisons only with TICA. It is possible to use NAUTICA with any other framework for predicting interaction, and TACO and CENDIST are just examples. However, we did not use them in this work (see changes at the beginning of the* *Discussion**Section)*.

*The choice of focusing only on TICA was dictated by two main factors: first of all, the CENTDIST software is not available anymore (the URL provided in the original manuscript* *http://compbio.ddns.comp.nus.edu.sg/~chipseq/centdist/**is not reachable on 07 Feb. 2020); TACO is still available but we cannot guarantee any maintenance of its software. Secondly, we plan to release our software either as a stand-alone package or as a web application, and in both cases we do not have the ownership of the two aforementioned tools.*

7) ---(p17 l24)--- "We estimate the tail of the distribution (viz., the portion of the distribution which is significantly different from the rest) to start at N12=10. Thus, we collapse the tail and split the PPI shared-interactor distribution into eleven bins: N12 = 0, N12= 1, …, N12 = 9, and N12=10+." What statistical method was used to assess significance?

*RESP: There is no standard definition of the right tail of a distribution; rather than using quantiles, we empirically observed that, if we consider nodes with N12 less than 10, then there is a large drop in their number. Thus, 10 appears a good threshold to delimit the right tail in the N12 distribution.*

8) ---(p22 l31)--- "There are too few COMP cases to form a null distribution of their own. However, we believe this distribution is close to the NINT null distribution, so we also use the NINT null distribution to weight TF pairs curated as COMP interactions." Authors should also present the theoretical considerations behind the assumption that the COMP and NINT distributions are close to each other.

*RESP: Our theoretical considerations were added to the text (see “Methods: Recall calibration and evaluation” Section)*

Minor comments

1) -------------- The authors often use the shared edges, shared connections, shared interactions expressions with the same meaning as shared neighbor, shared interactor, shared partner, however from network point this two expression groups describes different objects. The first group of expressions corresponds to edges that directly connect the two nodes in question, while the second delineates a topology with a third node which is connected to both investigated nodes by discrete edges. In the manuscript the expressions from the first group is often used in inappropriate context that can be confusing for the readers.

2)---(p6 l8)--- "*τH* = 8 and *τH* = 5." Both are TH. I think the correct form is: TH = 8 and TL = 5

3)---(p14 l11)--- "...this last group can be found in the bottom left branch of Fig. 3," Fig. 4,

4)---(p16 l23)--- "We restrict our attention to edges that are TFs and have degree at least equal to 3 in the full network." We restrict our attention to nodes...

5)---(p17 l11)--- "Then N12 can be thought of as the number of coworkers that two colleagues have." Then N12 can be thought of as the number of shared coworkers that two colleagues have.

6)---(p30 l13)--- " "Figure 3 Distribution of the number of shared interactions (“co-citations”) between TFs in the human, physical, multi-validated BioGRID network." In the text the "co-citations" example was appropriate which well explained and illustrated the network topology of interest, however mentioning it in the figure legend is rather confusing as no co-citation data is plotted in Fig. 3.

7)-------------- The citation of the TICA publication appears in the paper in a truncated form under reference number 2.

8)-------------- Resolution of Fig. 4 is poor.

9)-------------- There are no axis labels on charts shown in Fig. 5 and Fig. 6.

*RESP: All minor comments (1-9) were addressed*

10) -------------- Table ST2 What sort of data the PID_NUM column contains? What KO means in manual_validation column?

*RESP: PID_NUM corresponds to the number of PUBMED ID pertaining to papers that cite both transcription factors. It has been renamed to PID_NUM_CO-CITED for clarity. KO means that the authors could not confirm by reading through the specific papers that the interactions belong to the corresponding label (COOP or COMP). This is most often due to the fact that the papers mention the interactions but do not provide evidence either way.*

2) Endre Barta

Reviewer Recommendation Term: Reject as unsound science

Quality of written English: Acceptable

Reviewer summary: The manuscript by Perna et al describes a new method aiming at classifying transcription factor interactions into three categories (COOP, COMP, NINT). The whole project is the extension of the authors’ previous TICA framework. The idea that using the BioGRID protein-protein interaction data allows the annotation of interaction types of co-localized proteins in the DNA is new and promising. For this annotation, the TICA framework provides the list of protein couples. Despite the novelty of the method and the intriguing promise of knowing the nature of interactions of two proteins localizing to the same spot on the DNA, the manuscript in this form is not suitable for publication in the Biology Direct.

Reviewer recommendations to authors

There are serious conceptual problems in the manuscript. Some of them originated from the TICA framework. The manuscript (and the TICA webpage) speaks about transcription factors, but in my view TF is a protein that has its cognate binding site on the DNA and it binds specifically to it (https://en.wikipedia.org/wiki/Transcription_factor). SMC3, EP300, HDAC1 and many others in the TR-TS lists are not transcription factors. It is an important distinction because they do not bind directly to the DNA, thus they cannot compete with a real TF for the binding to the DNA double helix. Also, their positions in the DNA not necessarily depend on a TF, which is already bound to the DNA double helix. In case of TF couples we may also speak about different scenarios. For example, there are composite elements like the DR1 (PPARg and RXRA) or the GATA-1 TAL1. PPARg and RXR recognize the same DR element, therefore if only one DR element is present or the spacer is different (like at DR5), they may compete for this binding site, but otherwise they bind to the DR1 composite element cooperatively. There are transcription factor families, which recognize the same consensus sequences. A good example is the E-box family or the nuclear receptors. The members of these families may compete for their cognate binding sites if the given TFs are expressed and present in the nucleus at the proper form. There are transcription factor binding sites, which are close together to be involved in the analysis but far enough for avoiding the direct physical contact between the bound proteins. It is not written in the manuscript, but at the TICA website as much as 2200 bp is allowed for the maximum distance between the couples. Is it possible to have any physical interaction between two TFs sitting on the DNA in 2000 bp distance? The described method should have analyzed separately the competitions where two TF compete for the same binding site, and where two proteins compete for binding to a third (TF) protein. The BioGRID annotations are coming from different articles. They are sometimes very old, many times they represent a very different situation and many times they are from a different organism. I do not think that these data are suitable for inferring general rules. Of course, in many cases the data from BioGRID can be applied for certain protein couples (like those that were specifically mentioned in the manuscript), but surely not in all cases. The method described in the manuscript raises questions as well. Basically, it is not convincing enough that there were any benefits against just considering the shared and direct interactions from the BioGRID. Both the TR and TS are built from the same data trio (interaction prediction labels, BioGRID edge extraction and the number of shared interactions). Among these the most important is the number of the shared interactions, which is just a number, therefore the authors can operate only with their distributions in the TR for deciding the thresholds. The BioGRID annotations are heavily biased. For example, proteins that have medical importance and thus they are more frequently studied, will have more interactions than the others. The authors provide only one “external” evidence. Namely, they show that the COOP cases are over-represented in the CORUM complexes. It is convincing, but one should also consider that most likely the CORUM database uses the same publications as the BioGRID. It is not clarified why the distance values between the couples are not used in the analysis. If two proteins on the DNA are closer to each other (in bp), they are more likely interacting to each other. Of course, it also depends on whether they are both TFs or not, or whether they have similar TFBS etc.

*RESP: We thank the reviewer for the detailed analysis of our paper. In particular, we acknowledge that the reviewer used a stricter definition of Transcription Factor than the one we used in our work. We added a paragraph in our manuscript clarifying we include proteins that bind other TFs in binding pockets. We also incorporated the points above as caveats in the Discussion section.*

*The reviewer’s comments seem to imply that interaction prediction and BioGRID edge existence are not as important as N*_*12*_*. This impression is incorrect. The interaction prediction label is in some cases critical for correct label prediction (for instance SPI1/TBP, a known cooperation case with 0 reported edges in BioGRID. If NAUTICA did not use the TICA prediction label, it would be mispredicted as a competition or noninteraction.)*

*We agree that CORUM might have bias towards well-studied proteins. Nevertheless, we have not found a better substitute. We further provide evidence (albeit potential bias towards well-studied proteins remains) additional to CORUM by manually curating predicted interaction using existing literature (subsection* ***Investigation of significant cases shows both novel and known interactions****), which is both external and not related to CORUM.*

*On the reviewer’s comments that the distance values between the couples are not used, it is actually not entirely correct. Recall that NAUTICA takes TICA’s predictions as its starting point. TICA makes its predictions on interaction between transcription factors by doing statistical tests on the distribution of the distance between couples. Therefore, NAUTICA actually uses the distance between couples, albeit indirectly.*

Minor issues

It is not clear whether the TICA interactions were from only promoter regions or also from enhancers. Did the authors consider the long-range interactions (where two TFs are bound to the DNA far from each other but interacting directly or through other proteins via a chromatin loop)? The TICA data are restricted for three cell lines but the BioGRID annotations based on experiments also from other types of cells or from in vitro data. It seems (although it is not totally clear) that in the initial decision tree all the TICA cases are considered not only those where there is a topological association. Similarly, couples missing from the TICA list were analyzed in the training set as well. Why is it necessary to use TICA at all (considering also that the distances between the couples are not used in the analysis)? Is it possible that two protein being very close together on the DNA and they are not interacting at all (annotated as NINT)? Relying on the ratio of the COOP, COMP and NINT cases at the TR and TS seems to be arbitrary. How would we know the in vivo ratios in the cells? The manuscript is too long and it is very difficult to read and understand. Some important information are missing. For example, nothing is mentioned about the nature of the TICA input data and no supplementary table for it. There is a training set and a test set available as a supplementary table, but no list, no statistics about the result from the whole TICA data. In this form, the described method is not reproducible. Numbering the pages could help a lot during the reviewing.

*RESP: We recommend the reader reviews the TICA paper for a correct understanding of the methodology. In* ***TF-TF interaction prediction****, we point out the following: “TICA is based on the concept of minimal distance couple. Briefly, binding sites from ENCODE narrowPeak datasets are reduced to their 1bp point source, and their positioning is compared across promoters of active genes.” We agree that we are summarizing TICA as opposed to explaining its details. We do this because the manuscript is already very dense (as the reviewer points out) and TICA is already described in its own paper.*

*We emphasize here that, by using the TICA interaction label in the model as a feature, we are using (an aggregation of) the distance between binding site couple (see previous response). Also, we do not use couples in the training and test datasets where TICA data is not available (cf.* ***Model training*** *– “To fit the two thresholds […] sampling the list of possible TF pairs for which data is available.”). We acknowledge that this might not be evident from the wording, so we added a clarification.*

*To the best of our knowledge there is no compiled resource that reports an estimation of the ratio of COOP, COMP, and NINT in in-vivo cells. We make a hopefully not-too-wild guess of this ratio. We will be most glad to use better information if we learn of any. In any case, it is easy to show that when NINT:COOP:COMP is y:x:x where y + 2x = 1, the precision is at least two folds better than random when x < 0.14 (i.e. when no more than 28% of random TF pairs are COOP or COMP, which seems a safe assumption); and this improvement monotonically increases as x decreases.*

## Supplementary information

**Additional file 1: Figure S1.** Comparison between relative risk and odds ratio in *N*_*12*_ distribution bins. **A.** Relative risk (RR) distribution of COOP vs NINT TF pairs in TR with respect to bin sizes. Each histogram reports the absolute value. **B.** Similar chart for odds ratio (OR).

**Additional file 2: Table ST1.** Listing of datasets TR and TS

**Additional file 3: Table ST2.** List of manually validated and novel NAUTICA predictions with highest N_12

**Additional file 4: Table ST3.** *χ*^2^ and *P* values according to bins for COOP versus NINT relative risk / odds ratio analysis.

## Data Availability

The datasets generated and/or analysed during the current study are available in the ENCODE repository, https://www.encodeproject.org/datasets/.

## Abbreviations

ChIP-Seq: Chromatin Immunoprecipitation followed by sequencing
COOP: Co-operations
COMP: Competitions
NINT: Noninteraction
PPI: Protein-protein interaction
TF: Transcription Factor
