# Supplementary material for: NAUTICA: classifying transcription factor interactions by positional and protein-protein interaction information
Source: Biol Direct. 2020 Sep 16;15:13. doi: 10.1186/s13062-020-00268-1 (PMC7493360; doi:10.1186/s13062-020-00268-1)
Supplement: Supplementary file 1 — Additional file 1: Figure S1. Comparison between relative risk and odds ratio in N12 distribution bins. A. Relative risk (RR) distribution of COOP vs NINT TF pairs in TR with respect to bin sizes. Each histogram reports the absolute value. B. Similar chart for odds ratio (OR). [file 13062_2020_268_MOESM1_ESM.docx]

**File S1 Additional discussion on relative risk/odds ratio analysis**

This file compliments the main text by presenting the relative risk and odds ratio analysis used to further support the usage of N12 as a feature, …

**Relative risk and odds ratio analysis**

It is natural to consider the output of TICA (or other tools that predict TF-TF interactions) and the existence of a direct edge in BioGRID as features for NAuTICA. On the other hand, the number of shared interactors is a less-known feature, the power of which as a measure of the co-operation level requires a deeper analysis. Two indicators (*relative risk*, RR; and *odds ratio*, OR) are computed for each bin of the PPI shared-interactor distribution (cf. Figures 5 and 6 in the main text).

The relative risk of two labels *L_1_* and *L_2_* in bin *i* is defined as

$RR_{i}(L_{1},L_{2}) = \frac{P(L_{1}\in i)}{P(L_{2}\in i)}$,

where the numerator is the ratio between the number of pairs predicted as *L_1_* in bin *i* and the total number of *L_1_* pairs, and the denominator is computed similarly with respect to *L_2_*. Whereas, the odds ratio of two labels in a bin is instead defined as between the ratio of *L_1_* pairs to *L_2_* pairs in bin *i* and the ratio of *L_1_* to *L_2_* pairs *not* in bin *i*, for any given *i*. We compute each one of RR and OR in three cases: co-operation vs non-interaction, co-operation vs competition and competition vs non-interactions.

For example, the following rules can be derived from RR and OR: if $RR_{i}(COOP,NINT) > 1$, then COOP TF pairs have higher preference for bin *i* (compared to other bins) than NINT TF pairs; if $OR_{i}(COOP,NINT) > 1$, then it is more likely to see COOP TF pairs in bin *i* (compared to other bins) than NINT TF pairs; both of these can be similarly defined for different labels $(L_{i},L_{j})$. We computed the OR and RR graphs for co-operation (COOP) versus non-interactions (NINT) for all interactions in our training dataset TR, shown in Figure S1. We observe that both measures are much higher than 1 in bins 8, 9, and 10+ and less than 0.5 in bin 0, suggesting that our use of high *N_12_* as a marker for co-operation is sound. We also note that bin 5 is enriched, further supporting our choice of $\tau_{H} = 5$.

We also ran a $\chi^{2}$significance test on the counts used to compute the odds ratio and relative risk, to assess whether the results are significant. Results in Table S1. Bins 0, 8, 9 and 10+ are significant with respect to the $\chi^{2}$ statistic, providing additional support to our decision.
